# Inhibition of Bruton Tyrosine Kinase Reduces Neuroimmune Cascade and Promotes Recovery after Spinal Cord Injury

**DOI:** 10.3390/ijms23010355

**Published:** 2021-12-29

**Authors:** Chen Guang Yu, Vimala Bondada, Hina Iqbal, Kate L. Moore, John C. Gensel, Subbarao Bondada, James W. Geddes

**Affiliations:** 1 Spinal Cord and Brain Injury Research Center, Department of Neuroscience, College of Medicine, University of Kentucky, 741 S. Limestone Street, Lexington, KY 40536, USA; vbond0@uky.edu (V.B.); hina.iqbal@uky.edu (H.I.); km00800@gmail.com (K.L.M.); 2 Department of Physiology, College of Medicine, University of Kentucky, 741 S. Limestone Street, Lexington, KY 40536, USA; gensel.1@uky.edu; 3 Department of Microbiology, Immunology & Molecular Genetics, Markey Cancer Center, College of Medicine, University of Kentucky, 800 Rose St, Lexington, KY 40536, USA; subbarao.bondada@uky.edu

**Keywords:** Bruton tyrosine kinase, Ibrutinib, neuroimmune, spinal cord injury, locomotion, neuroprotection, B cells, glial cells

## Abstract

Microglia/astrocyte and B cell neuroimmune responses are major contributors to the neurological deficits after traumatic spinal cord injury (SCI). Bruton tyrosine kinase (BTK) activation mechanistically links these neuroimmune mechanisms. Our objective is to use Ibrutinib, an FDA-approved BTK inhibitor, to inhibit the neuroimmune cascade thereby improving locomotor recovery after SCI. Rat models of contusive SCI, Western blot, immunofluorescence staining imaging, flow cytometry analysis, histological staining, and behavioral assessment were used to evaluate BTK activity, neuroimmune cascades, and functional outcomes. Both BTK expression and phosphorylation were increased at the lesion site at 2, 7, 14, and 28 days after SCI. Ibrutinib treatment (6 mg/kg/day, IP, starting 3 h post-injury for 7 or 14 days) reduced BTK activation and total BTK levels, attenuated the injury-induced elevations in Iba1, GFAP, CD138, and IgG at 7 or 14 days post-injury without reduction in CD45RA B cells, improved locomotor function (BBB scores), and resulted in a significant reduction in lesion volume and significant improvement in tissue-sparing 11 weeks post-injury. These results indicate that Ibrutinib exhibits neuroprotective effects by blocking excessive neuroimmune responses through BTK-mediated microglia/astroglial activation and B cell/antibody response in rat models of SCI. These data identify BTK as a potential therapeutic target for SCI.

## 1. Introduction

Traumatic spinal cord injury (SCI) impacts motor, bowel, bladder, and sexual function, resulting in a tremendous socioeconomic impact on affected individuals and the health care system [1,2]. In the United States, there are over 17,000 new injuries each year [3], with approximately 300,000 persons living with an SCI [4], based on SCI incidence and prevalence and extrapolating from the 2020 census. Current treatments for acute SCI are largely limited to stabilizing the spine and providing palliative care. No approved therapies are available for reducing motor impairment, bladder dysfunction, and other deficits.

Over the past two decades, it has become evident that SCI elicits multicellular and sequential acute inflammatory and delayed autoimmune responses which include activation of microglia, macrophages, and astrocytes, along with B lymphocytes [5,6,7,8,9,10,11]. B cells play a central role in the adaptive immune system and autoimmunity, while microglia, macrophages, and astrocytes are key mediators of the innate immune system and inflammatory response. Although the inflammatory and immune systems can be neuroprotective and growth promoting, their excessive activation shifts the pendulum towards pathology and contributes to neurodegeneration and resultant functional deficits following SCI. 

The inflammatory response within the first few days post-injury consists of activation of pro-inflammatory M1-microglia, macrophages, and astrocytes that trigger activation of NLRP3 inflammasomes and production of oxidative enzymes (NOX2) and pro-inflammatory cytokines (IL-1β, TNF-α, IL-6, IL-18,) [6,12,13,14,15,16,17,18,19,20,21,22]. Contusive SCI also activates B cells over days to months. Autoreactive B cell activation contributes to plasma cell formation to produce autoantibodies, causing axon/myelin damage [23,24,25,26,27,28,29,30,31]. The autoimmune and inflammatory cascades exacerbate spinal tissue/axon damage, locomotor deficits and bladder dysfunction [12,16,17,24,32,33,34,35,36]. As the inflammatory/immune cascades have both pathogenic and protective roles after SCI, a challenge is to reduce the pathogenic autoimmune and pro-inflammatory cascades and promote functional recovery following SCI without creating immunodeficiency.

Evidence is accumulating that Bruton’s tyrosine kinase (BTK) is a key regulator of the innate and adaptive immune systems. However, this is largely based on findings in non-CNS injury and autoimmune disorders such as lupus, rheumatoid arthritis, and B cell malignancies [37,38,39,40].

BTK was originally identified as the gene mutated in X-linked agammaglobulinemia (XLA) and was subsequently shown to be the rate-limiting step in B cell receptor signaling and B cell survival and differentiation [41,42]. BTK links B cell receptor activation to B cell survival through phosphorylation (activation) of BTK at Y551 by Src family kinases including Syk (spleen tyrosine kinase) and autophosphorylation at Y223 [43]. Phospho-BTK activates NF-κB pathways, leading to gene transcription, B cell proliferation and differentiation into plasma cells. Thus, BTK is an important mediator of the B cell component of the adaptive immune response [44]. BTK is also present in myeloid cells including microglia, macrophages, and neutrophils—components of the innate immune/inflammatory response in the CNS [45,46]. BTK inhibition reduces the inflammatory response in a range of conditions including pneumonia, arthritis, ischemic brain injury, and lipopolysaccharide-induced inflammation [47,48,49,50,51,52].

The objective of this study was to evaluate the neuroprotective effects of BTK inhibition against the pathogenic neuroimmune injury cascades, tissue damage, and locomotor deficits following SCI. The BTK inhibitor used, Ibrutinib (Imbruvica^®^, previously PCI-32765), is a first-in-class irreversible inhibitor of BTK and is FDA approved for chronic lymphocytic leukemia [53]. Ibrutinib forms a covalent bond with Cys-481 in the ATP binding site of BTK (Cys-483 in rat BTK).

## 2. Results

No significant differences in actual force, displacement, or velocity were found between Ibrutinib-treated and vehicle-treated groups, indicating similar injuries to all animals (Table 1). Ibrutinib treatment was well tolerated and did not result in alterations in body weight, as compared to vehicle-treated animals, following SCI (Table 2). Bladder infection, detected by examining urine color (green-yellow) and transparency (cloudy) and hematuria, were not observed in any of the rats. Similarly, we did not observe bleeding in the eyes, forelimbs, and hindlimbs.

### 2.1. BTK Upregulation and Phosphorylation (Activation) following SCI, and Inhibition by Ibrutinib

**Figure 1:**
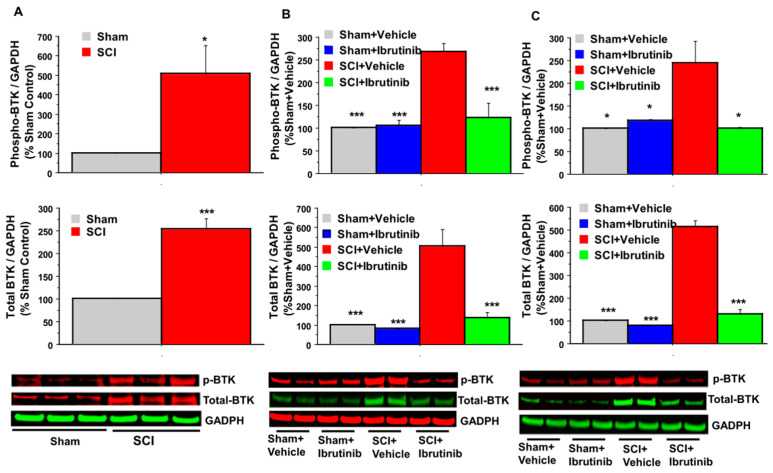
Effects of SCI and Ibrutinib treatment on BTK phosphorylation and total BTK protein analyzed by quantification of Western blotting data. Western blot analysis of spinal cord samples (60 μg of protein extract each sample) at lesion epicenter showed that contusion injury increased BTK phosphorylation and total BTK protein in the spinal cord 2 (**A**), 7 (**B**), and 14 (**C**) days post-injury compared with sham-operated animals. Ibrutinib treatment 6 mg/kg/day, Scheme 3 h post-injury for 7 and 14 days resulted in reduced levels of BTK phosphorylation and total BTK protein in the spinal cord lesion site at 7 (**B**) and 14 (**C**) days after contusive SCI compared with vehicle-treated animals. Quantification of total BTK/GADPH or phospho-BTK/GAPDH (%Sham control for 2-day time point and %Sham + Vehicle for 7 and 14 days- time points) after contusive SCI was performed by the fold of blot density (GAPDH as loading control). Antibodies were specific for the total BTK or BTK phosphorylation (phospho-BTK-Y223). Data are presented as mean ± S.E.M. (for 7-day time point, *n* = 4 per group, for 14-day time point, *n* = 2 per group) and analyzed with one-way ANOVA followed by Bonferroni post hoc analysis, (**B**,**C**): * *p* < 0.05, *** *p* < 0.001 compared to vehicle-treated SCI animals. (**A**): * *p* < 0.05, *** *p* < 0.001, compared to sham control, *t*-test, *n* = 3 per group.

Following contusive SCI (180 kdyn, T10) produced using the Infinite Horizons (IH) Impactor in female Sprague-Dawley (SD) rats, age three months, western blot analysis of the spinal cord at the lesion site revealed that BTK expression was elevated 2.5-fold at two days post-SCI, and 4–5-fold at 7- and 14-days post-injury (Figure 1). BTK phosphorylation at Y223, indicative of activation, was increased 5-fold at two days post-SCI, and by approximately 2.5-fold at 7- and 14-days post-injury, as compared to sham or sham + vehicle treatment groups. 

Ibrutinib (6 mg/kg/day, IP, starting 3 h post-injury for 7 and 14 days) prevented the SCI-induced elevation in total BTK and pBTK at 7 and 14 days after SCI in rats compared with vehicle-animals (Figure 1). Levels of both total BTK and pBTK were not significantly different in the SCI + Ibrutinib and the sham injury groups.

### 2.2. BTK Inhibition with Ibrutinib Treatment Reduces Plasma Cell Formation and Antibody Production in the Injured Spinal Cord

**Figure 2:**
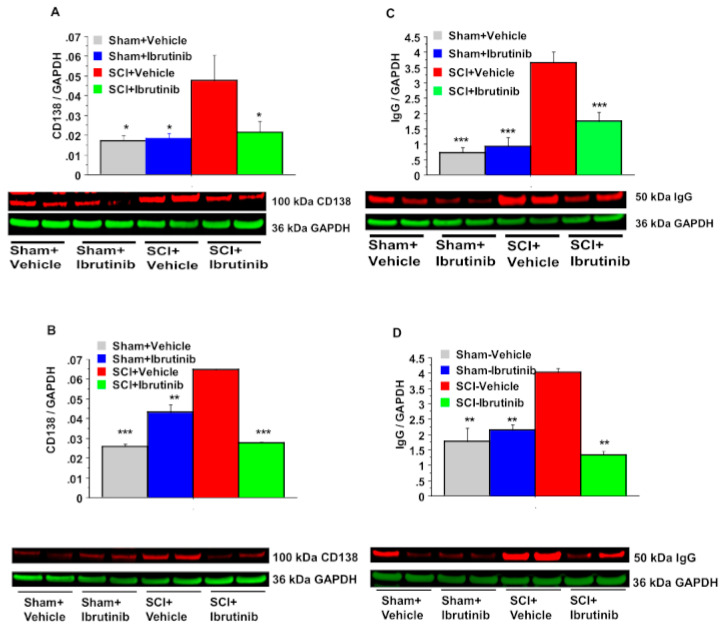
Effects of Ibrutinib post-treatment on levels of CD138 and total IgG. analyzed by quantification of Western blotting data. Western blot analysis of spinal cord samples (60 μg of protein extract each sample) at lesion epicenter showed that contusion injury increased CD138 (**A**,**B**) and total IgG (**C**,**D**) in the spinal cord 7 (**A**,**C**) and 14 (**B**,**D**) days post-injury compared with sham-operated animals. Ibrutinib treatment (6 mg/kg/day, starting at 3 h post-injury for 7 days) resulted in reduced levels of CD138 (**A**,**B**) and total IgG (**C**,**D**) in the spinal cord lesion site at 7 (**A**,**C**) and 14 (**B**,**D**) days after contusive SCI compared with vehicle-treated animals. Quantification of CD138/GADPH and total IgG/GAPDH 7 and 14 days after contusive SCI was performed by the fold of blot density. Data are presented as mean ± S.E.M., *n* = 4 per group (7-day time point) or *n* = 2 per group (14-day time point), and analyzed with one-way ANOVA followed by Bonferroni post hoc analysis, * *p* < 0.05, ** *p* < 0.01, *** *p* < 0.001, compared to vehicle-treated SCI animals.

In the same rat SCI model (T10, 180 kdyn), SCI resulted in elevated levels of CD138 (syndecan 1) by 2.6-fold at both 7 (Figure 2A) and 14 (Figure 2B) days post-SCI. CD138 is a marker of Ig-producing plasma cells. Ibrutinib treatment markedly reduced the SCI-induced elevation in CD138 levels at both post-injury time points. Similar to CD138, SCI resulted in elevated IgG levels at the injury epicenter. IgG levels were increased 4.7-fold at one week (Figure 2C), decreasing to 2.2-fold by two weeks (Figure 2D) post-SCI. Ibrutinib treatment prevented the elevations in IgG at both post-injury time points.

### 2.3. Ibrutinib Did Not Result in Reduced Levels of Splenic B Cells

**Figure 3:**
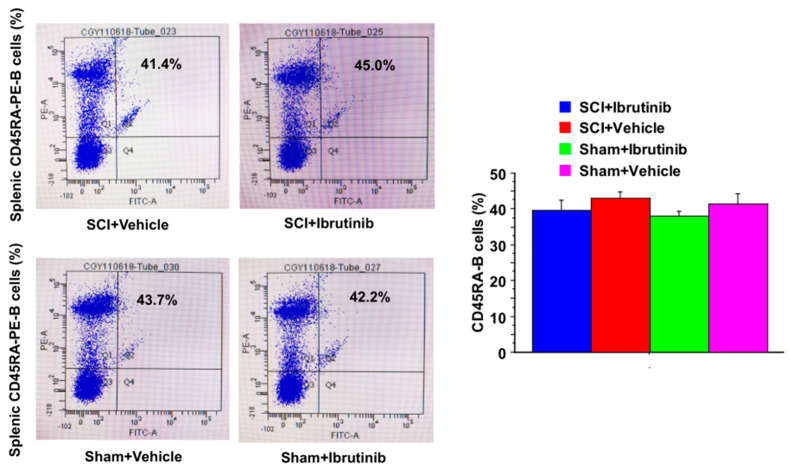
Ibrutinib did not reduce CD45RA-positive B cell population after SCI Injury. Flow cytometry analysis of splenic samples showed that there were no differences in CD45RA-positive B cell population between Ibrutinib-treated and vehicle-treated injured or sham-operated animals at 1-week post-injury. Anti-CD45RA-PE antibody was specific for the rat CD45RA-B cells. Data are presented as mean ± S.E.M., *n* = 4 per group, and analyzed with one-way ANOVA followed by Bonferroni post hoc analysis.

Flow cytometry showed no significant difference in normal splenic CD45RA+ B cell numbers between Ibrutinib and Vehicle groups at seven days post-injury (Figure 3). This data suggested that Ibrutinib did not result in reduced levels of splenic normal CD45RA+ B cells. 

### 2.4. Active Microglia/Macrophages or Astrocytes Express Phospho-BTK following Acute SCI in Rats

**Figure 4:**
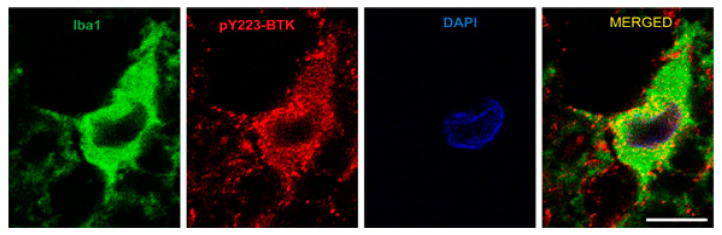
Phospho-BTK is localized to microglia 3 days following SCI, measured by immunofluorescence imaging. Photomicrographs of representative transverse spinal sections taken from spinal cord injured rat. Double immunofluorescent imaging analysis of spinal cord sections at lesion site showed phospho-BTK (red) expressed in microglia (Iba1, green) 3 days post-injury. The sections were immunostained with a primary antibody against phospho-BTK (red), and Iba1 (green) and counterstained with DAPI to identify cell nuclei. Phospho-BTK immunostaining was uniformly observed in cells immunoreactive for Iba1 (a specific calcium-binding protein for activation of microglia and macrophages [54]). This is from 3 days post SCI, at which time the Iba1 cells are predominantly microglia. The SCI conditions are as described in other figures. The images were obtained from the ventral white matter, in a section rostral 1 mm from the lesion epicenter using the Nikon confocal microscopy system (Nikon C2+, Melville, NY, USA) in the Spinal Cord and Brain Injury Research Center imaging core at the University of Kentucky. Scale bar: 10 μm.

Double immunofluorescent imaging analysis of spinal cord sections near the lesion epicenter demonstrated that pBTK (red) is expressed in cells immunoreactive for Ionized calcium-binding adaptor molecule 1 (Iba1) (green, Figure 4). However, it was not demonstrated that pBTK co-localized with cells expressing glial fibrillary acidic protein GFAP (results not shown). Iba1 is a microglia/macrophage-specific calcium-binding protein [54] whose expression is upregulated in activated microglia where it contributes to phagocytosis [55,56]. This is from three days post-SCI, at which time the Iba1 cells are predominantly microglia [57]. The images were obtained from the ventral white matter, in a section rostral 1 mm from the lesion epicenter. In the absence of primary antibody, faint green immunofluorescence was observed but this was distinct from that observed for Iba1 or GFAP immunoreactivity. Immunofluorescence in the red channel was not observed in the absence of the primary antibody.

**Figure 5:**
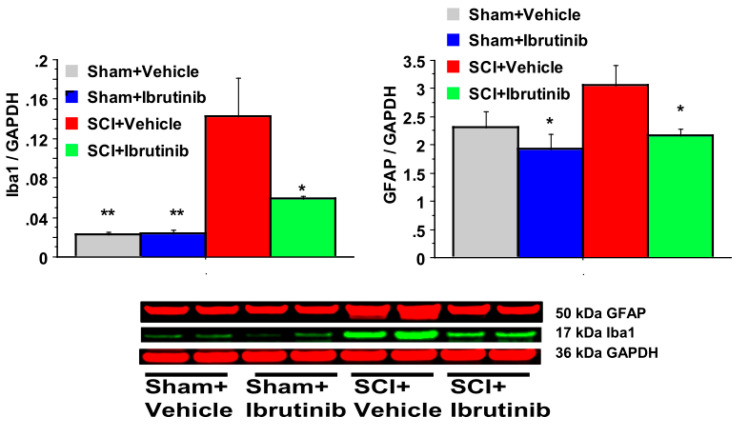
Effects of Ibrutinib post-treatment on Iba1 and GFAP proteins analyzed by quantification of Western blotting data. Western blot analysis of spinal cord samples (60 μg of protein extract each sample) at lesion epicenter showed that contusion injury increased Iba1 and GFAP activity in the spinal cord seven days post-injury compared with sham-operated animals. Ibrutinib treatment (6 mg/kg/day, starting at 3 h post-injury for seven days) resulted in reduced levels of Iba1 and GFAP in the spinal cord lesion site at 7 days after contusive SCI compared with vehicle-treated animals. Quantification of Iba1/GADPH and GFAP/GADPH seven days after contusive SCI was performed by the fold of blot density. Antibody was specific for the Iba1 or GFAP. Data are presented as mean ± S.E.M., *n* = 4 per group, and analyzed with one-way ANOVA followed by Bonferroni post hoc analysis, * *p* < 0.05, ** *p* < 0.01 compared to vehicle treated SCI animals.

SCI resulted in a dramatic 7-fold elevation in Iba1 levels at seven days post-injury (Figure 5). This increase was reduced, but not abolished, following treatment with Ibrutinib. 

SCI resulted in a modest elevation in GFAP levels at the 7-day time point, which was prevented by Ibrutinib treatment.

### 2.5. BTK Inhibition with Ibrutinib Treatment Reduces Activation of Microglia/Macrophages and Astrocytes following SCI in Rats

Western blot data demonstrated that SCI resulted in elevated levels of Iba1 (marker for microglia and macrophages) and GFAP (astrocytes) at seven days post-injury (Figure 5). Ibrutinib treatment for seven days post-injury attenuated the injury-induced elevations in Iba1 and GFAP at seven days post-injury (Figure 5). 

### 2.6. BTK Inhibition with Ibrutinib Treatment Improves Functional Outcomes

**Figure 6:**
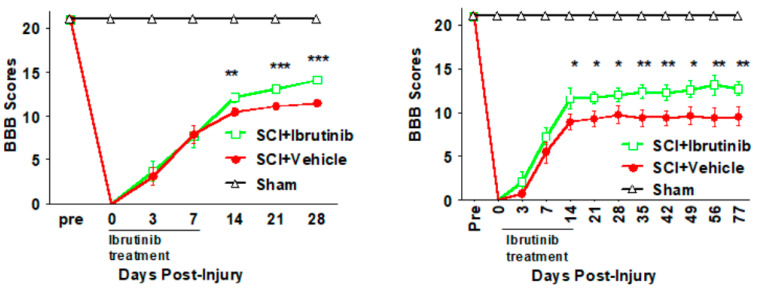
Effects of Ibrutinib post-treatment on locomotor function after SCI. (**Left panel**): Acute treatment with Ibrutinib improved locomotor function after SCI. The 6 mg/kg/day of seven days IP treatment with Ibrutinib resulted in improved locomotor function, measured by BBB scores, up to 28 days after contusive SCI compared to vehicle treated controls. (**Right panel**): Prolonged treatment with Ibrutinib for two weeks improved long-term locomotor function after SCI. The prolonged Ibrutinib (6 mg/kg/day for 14 days) resulted in long-term improved locomotor function, measured by BBB scores, up to 11 weeks after contusive SCI compared to vehicle-treated controls. Contusive SCI was produced using the Infinite Horizons impactor, 180 kdyn setting at T10. Data were presented as mean ± SEM and analyzed with repeated measures ANOVA followed by Bonferroni post-hoc analysis, * *p* < 0.05, ** *p* < 0.01, and *** *p* < 0.001, Ibrutinib treatment vs. vehicle treatment, *n* = 10 per group.

Locomotor activity was assessed using the BBB test for both the 7- and 14-day Ibrutinib treatment regimens. Following the 7-day treatment regimen, BBB scores were similar to vehicle-treated animals during the Ibrutinib treatment period. During the subsequent three weeks, BBB scores were improved in the Ibrutinib vs. vehicle treatment group (Figure 6 left panel). As the BBB scores were trending upwards at 28 days post-injury, we sought to determine if longer treatment would result in greater improvement and extended the time course of the locomotor function assessment. Following SCI and 14 days of Ibrutinib treatment, the BBB profile out to 28 days was similar to that observed with 7 days of Ibrutinib, with improvements noted in the Ibrutinib treatment group at 14–28 days post-injury. Similar BBB scores persisted until 11 weeks (77 days) post-SCI, as plateaus were observed for both the vehicle- and Ibrutinib-treatment groups (Figure 6 right panel).

**Figure 7:**
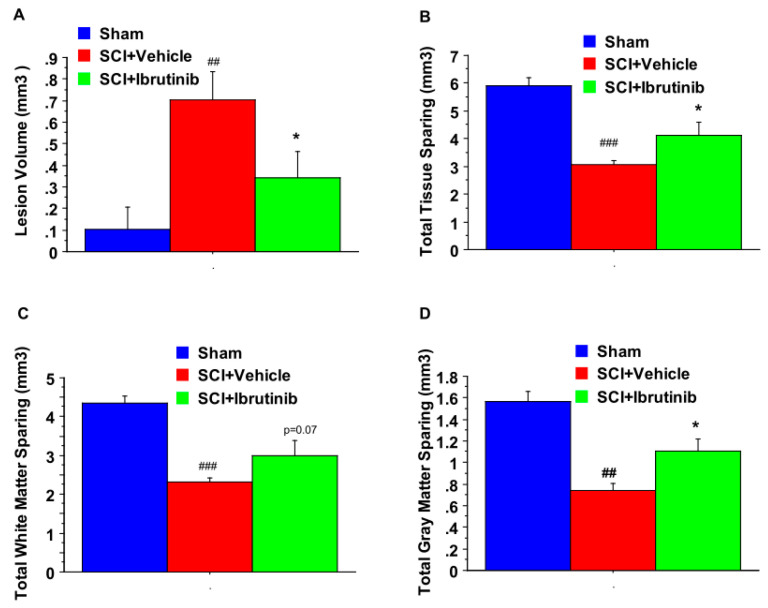
Effects of the 2-week treatment with Ibrutinib on lesion volume and total tissue sparing after SCI. Ibrutinib post-treatment (6 mg/kg/day, IP, starting at 3 h post-injury, daily for two weeks) resulted in a significant decrease in lesion volume (**A**), and a significant improvement in total tissue sparing (**B**). The increase in total white matter sparing following Ibrutinib treatment did not reach statistical significance (**C**). Ibrutinib increased total gray matter sparing (**D**) after contusion injury to the spinal cord (T10, 180 kdyn) compared to vehicle controls. Data were presented as mean ± SEM and analyzed with repeated measures ANOVA followed by Bonferroni post-hoc analysis, * *p* < 0.05 (Ibrutinib treatment at 6 mg/kg/day vs. vehicle treatment), ## *p* < 0.01 & ### *p* < 0.001 (compared with Sham), *n* = 5/group.

**Figure 8:**
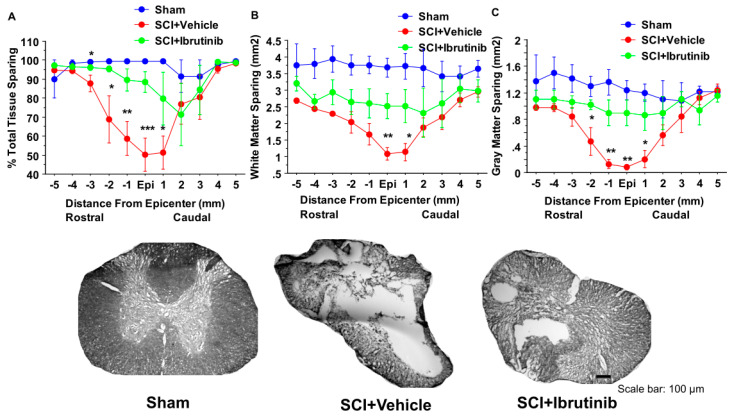
Effects of the 2-week treatment with Ibrutinib on lesion epicenter and spread of tissue damage after Scheme 2. Two-week treatment with Ibrutinib resulted in significant increases in tissue-sparing at the epicenter, 1, 2, & 3 mm rostral to the injury epicenter, and 1 mm caudal to the lesion epicenter (**A**), white matter sparing at lesion epicenter and 1 mm caudal to the lesion epicenter (**B**), and gray matter sparing at epicenter, 2 mm, 1 mm rostral and 1 mm caudal to the lesion epicenter (**C**) at 11 weeks following contusion injury to the spinal cord (T10, 180 kdyn). Data were presented as mean ± SEM and analyzed with repeated measures ANOVA followed by Bonferroni post-hoc analysis, * *p* < 0.05, ** *p* < 0.01, and *** *p* < 0.001, compared with vehicle treatment, *n* = 5/group. **Bottom Panel:** Photomicrographs of representative transverse spinal cord sections from rats at 11 weeks after contusive SCI (T10, 180 kdyn). The sections were from the lesion epicenter, obtained from a Sham (left), vehicle-treated injured rat (middle), and Ibrutinib (6 mg/kg/day)-treated injured rat (right). The sections were stained with eriochrome cyanine for myelin. Scale bar: 100 μm.

For the 14-day treatment group, we evaluated tissue sparing at the conclusion of the locomotor assessment period. Histological analysis of spinal cord sections showed that prolonged Ibrutinib treatment (6 mg/kg/day ip starting at 3 h postinjury for 14 days) significantly reduced lesion volume (Figure 7A) and improved total tissue sparing (Figure 7B), total white matter sparing (Figure 7C), and total gray matter sparing (Figure 7D) at 11 weeks post-injury. At the epicenter and 5 mm rostral and caudal to the epicenter, Ibrutinib treatment resulted in a significant increase in the tissue-sparing, white matter sparing, and gray matter sparing following contusion injury to the spinal cord (Figure 8, top panel). The spinal sections were stained with eriochrome cyanine for myelin (Figure 8, bottom panel).

## 3. Discussion

The goals of the present study were to (1) determine if BTK expression was altered and if BTK was activated following SCI; (2) to evaluate whether BTK inhibition would reduce B cell autoimmune and inflammatory responses following SCI; and (3) to examine if BTK inhibition would result in improved pathological and functional outcomes. This was evaluated using a rat T10 contusion injury model of moderate-severe spinal cord injury. 

The results demonstrate BTK activation (phosphorylation) and BTK protein upregulation at 2-, 7- and 14-days following SCI. Previously, we observed elevated BTK expression and BTK phosphorylation in SCI-injured rats at four weeks post-injury [17]. BTK was originally identified as the gene mutated in X-linked agammaglobulinemia (XLA) and was subsequently shown to be the rate-limiting step in B cell receptor signaling and autoreactive B cell survival and differentiation [41,42]. BTK is an important mediator of the autoreactive B cell component of the autoimmune response [44]. Thus, the upregulation and activation of BTK following SCI are hypothesized to contribute to pathogenic B cell activation and autoimmunity observed following SCI. 

Following low thoracic SCI in mice, B cells proliferate in bone marrow and spleen and then migrate to the injury site [9]. B cells are activated to form antibody-secreting plasma cells. At the injury site, B cells form structures similar to ectopic follicles and produce autoreactive immunoglobulins [9,24]. Antibody secreting B cells are found in the injured spinal cord and CSF, and antibodies isolated from injured mice cause pathology in naïve mice [24]. Autoantibodies and autoimmunity are also evident following human SCI [58,59,60]. We therefore sought to determine if BTK inhibition would alter levels of B cells, plasma cells, and immunoglobulins following SCI. 

Administration of Ibrutinib following SCI abolished the SCI-induced elevation of BTK and pBTK. Ibrutinib treatment also decreased levels of CD138, a marker of plasma cells [61], and reduced IgG levels following SCI. SCI did not increase, and Ibrutinib treatment did not reduce, numbers of splenic B cells at seven days post-injury. The effects of Ibrutinib on B cell activation (pBTK), plasma B cell (CD138), and IgG levels in the injured spinal cord are consistent with elevated BTK levels and activation following SCI promoting pathogenic B cell activation and autoimmunity [62]. However, the lack of change in splenic B cell levels following both SCI and Ibrutinib treatment was surprising. 

Splenic CD45R+ B cell levels are significantly elevated within seven days of a moderate severity contusive SCI at thoracic level T9 in mice, with levels peaking at 14 days and remaining elevated through 28 days post-SCI [9]. Following moderate severity contusive SCI in rats at T10, we previously observed a modest but significant increase in CD45RA+ B cells at 28 days post-injury [17]. The lack of significant increase in splenic CD45RA+ B cells at 7 days following SCI in the present study may reflect differences in the robustness of the B cell response to injury in mice vs. rats, the relatively early post-injury time point examined, or a combination of the above. In contrast to low thoracic injury, splenic B cell levels are decreased following clip-compression SCI in rats at C7-T1 and following higher thoracic injury (T3) in mice due to disruption of the sympathetic nervous system and resultant immunosuppression [63,64]. 

Ibrutinib treatment decreased levels of plasma cell marker CD138 and IgG in the injured spinal cord but did not alter splenic B cell numbers. Enhanced BTK activity is implicated in the breach of self-tolerance checkpoints in autoimmunity [65]. Overexpression of BTK in B cells results in germinal center and plasma cell formation, antinuclear antibody production, and autoimmune disorders [39,65,66,67]. Importantly, BTK appears to act as a rheostat and not as an on-off switch, with overexpression leading to autoimmunity while BTK inhibition improves outcomes of autoimmune disorders such as rheumatoid arthritis and multiple sclerosis [40,48,68]. In a BTK^lo^ lyn^−/−^ mouse model, mice with reduced levels of BTK expression did not develop autoantibodies but had normal B cell development [69]. Autoreactive B cells depend upon BTK for survival to a greater degree than normal B cells and BTK inhibition suppresses autoreactive B cell differentiation into plasma cells and autoantibody production without creating B cell immunodeficiency [40]. The reduction in CD138 and IgG immunoreactivity in the injured spinal cord following Ibrutinib treatment is consistent with BTK acting as a rheostat in B cell activation and development and demonstrates that BTK inhibition may prevent the over-activation of B cells and the autoimmune response following SCI, while preserving normal levels of B cells. This contrasts with B cell-depletion via anti-CD20 antibodies, which suppresses both pathogenic and normal B cells after SCI [24,70,71], resulting in increased vulnerability to opportunistic infection due to immunodeficiency [28,72].

In addition to B cells, BTK is expressed in B cells and in cells of myeloid origin including macrophages, microglia, and neutrophils, components of the CNS innate immune system [47]. Originally thought to be non-functional due to the lack of B cell receptors in myeloid cells, BTK was subsequently shown to regulate activation of monocytes, macrophages, and microglia, with BTK deficiency resulting in reduced inflammatory responses [49,73,74]. Daily Ibrutinib treatment following SCI significantly attenuated the SCI-induced elevation in the microglia/macrophage marker Iba1 at the lesion site on the spinal cord seven days post-SCI, consistent with reduced microglial activation and macrophage infiltration. Ibrutinib also reduced the recruitment of neutrophils to the injured spinal cord at 24 h post-injury, following administration immediately after or 12 h post-injury, or both [75]. 

BTK deficient mice, and mice treated with Ibrutinib, exhibit decreased recruitment of M1 macrophages/microglia following intraperitoneal administration of lipopolysaccharide and also show increased expression of immunosuppressive M2-associated markers following M1 polarizing stimuli [52,76]. BTK blockade also reduces microglial phagocytosis in vitro and in vivo [74]. Together, these results demonstrate that post-injury administration of Ibrutinib attenuates the myeloid-cell mediated inflammatory response following SCI, reducing the activation of microglia and the infiltration of macrophages. BTK inhibition may also alter the phenotype of microglia and macrophages following SCI based on findings in other models of inflammation.

Although not myeloid cells, reactive astrocytes are increasingly recognized as a component of the innate immune response to CNS injury and of the adaptive immune response and autoimmunity [77,78,79,80,81]. In the initial stages, reactive astrocytes surround the lesion site and protect against the spread of injury. Later, hypertrophic astrocytes express pro-inflammatory factors and chondroitin sulfate proteoglycans (CSPGs), contributing to neurodegeneration and glial scar formation [17,82]. The present data demonstrated that Ibrutinib treatment reduced levels of the astrocytic marker glial fibrillary acidic protein at seven days following SCI. This is likely an indirect effect of Ibrutinib on microglia and macrophages, as activated microglia can induce astrocytes to become reactive and neurotoxic [20]. Previously, Ibrutinib administration in mice reduced both microglial and astrocyte activation following intraperitoneal injection of LPS in mice [52].

Based on the results of the present study showing that BTK signaling is critical to both B cell autoimmune and microglia/macrophage and astrocyte inflammatory responses after SCI, the third objective of the present study was to investigate whether post-injury administration of the BTK inhibitor Ibrutinib would reduce pathological and functional deficits after SCI in rats. Ibrutinib, administered IP at 6 mg/kg/day, beginning 3 h following contusive SCI followed by daily administration for two weeks, resulted in improved recovery of locomotor function and increased total tissue sparing, white matter sparing and gray matter sparing. 

Ibrutinib (Imbruvica^®^, previously PCI-32765), is a first-in-class irreversible inhibitor of BTK, forming a covalent bond with Cys-481 in the ATP binding site (Cys-483 in rat BTK). It is the most studied BTK inhibitor and is FDA approved for chronic lymphocytic leukemia [53]. Major adverse events include low platelet count, rash, diarrhea, and bruising (prescribing information for Ibruvica). It was also reported that there is an increased risk of bleeding and opportunistic infections. The latter two are of particular relevance to SCI. Bleeding is usually mild, rarely causes discontinuation of treatment, and results from multiple mechanisms including pre-treatment platelet levels [83,84]. With regard to infections, mechanisms implicated include thrombocyte impairment by Ibrutinib, but not other BTK inhibitors; off-target impairment of the ITK kinase expressed in T cells; and also impaired activation and M1 polarization of macrophages [53,85]. 

The off-target effects of Ibrutinib led to the development of second-generation BTK inhibitors with improved specificity [53,86]. We chose Tolebrutinib (SAR442168; PRN2246) for the proposed future studies. Tolebrutinib has improved penetration of the blood-brain barrier as compared to Ibrutinib, a greater affinity for BTK, and is in phase III clinical trials for primary progressive and relapsing multiple sclerosis [87,88] (Clinical trials NCT04410978; N CT04458051. Although Tolebrutinib has improved specificity for BTK as compared to Ibrutinib, it also inhibits TEC kinase, which also has Cys at a similar position in the ATP binding site, as do all irreversible BTK inhibitors [83]. Tolebrutinib did not result in bleeding in phase I trials [83,89]. 

In conclusion, the present study demonstrates that: (1) BTK activation and total BTK upregulation are implicated in neuroimmune pathogenesis of traumatic SCI and represent a promising therapeutic target; (2) BTK inhibition with Ibrutinib attenuates plasma cell formation and antibody production as well as activation of pro-inflammatory microglia, macrophages and astrocytes without causing a reduction of normal B cells; and (3) BTK inhibition with Ibrutinib treatment exhibits neuroprotective effects against tissue damage and locomotor deficits in rat models of SCI. Together, these results suggest the Bruton’s tyrosine kinase inhibitors as potential therapeutic agents for SCI.

## 4. Materials and Methods

### 4.1. Rigorous Experimental Design

Rats were randomly assigned to each group using Research Randomizer. All personnel who perform assessments were blinded to the treatment assignment. The sample size was determined, based on previous studies and power analysis to ensure sufficient statistical power.

### 4.2. Animals

Female Sprague–Dawley (SD) rats approximately three months of age, weighing 200–250 g, were used (Charles River, Indianapolis, IN, USA). Female rats are used due to the need for manual post-injury bladder expression, which is facilitated in females due to their shorter urethra. Given the neuroprotective effects of estrogen and progesterone and sex differences in many acute injury paradigms, it is essential to confirm efficacy in male rats in future studies. Rats were kept under standard housing conditions for at least one week following arrival in an enclosed, pathogen-free animal facility. All experimental procedures were approved and carried out in accordance with the Guidelines of the US National Institutes of Health and the Institutional Animal Care and Use Committee (IACUC) of the University of Kentucky.

### 4.3. Antibodies and Chemicals

Ibrutinib was purchased from MedChemExpress LLC (Monmouth Junction, NJ, USA). Anti-IgG and anti-Syndecan-1 (CD138, ab60199) antibodies were purchased from Abcam (Cambridge, MA, USA). Anti-phospho-BTK-Y223 antibody (5082), Total BTK (D3H3) antibody (5847), and anti-GFAP (D1F4Q)XP (12389) antibody were purchased from Cell Signal Technology. PE mouse anti-rat CD45RA antibody was purchased from BD Bioscience. Anti-GAPDH antibody and Iba1 antibody (SAB2702364) for Western blot were purchased from Sigma-Aldrich (St. Louis, MO, USA). Anti-Iba1 monoclonal antibody (MA5-27726), phosphor-BTK (Tyr223) polyclonal antibody (PA5-105619), goat-anti mouse Alexa Fluor 488 antibody (A-31620) and Donkey anti-rabbit Alexa Fluor 594 antibody were purchased from Fisher Thermo Scientific. Goat anti-mouse IR Dye 680 antibody, goat anti-rabbit IR Dye 680 antibody, goat anti-mouse IR Dye 800 antibody, and goat anti-rabbit IR Dye 800 antibody were purchased from Li-Cor.

### 4.4. Contusional SCI

SCI was modeled in rats using a moderately severe contusion injury (180 kdyn, T10, Infinite Horizon SCI Impactor) [17]. The contusive rat thoracic SCI is widely used and produces similar morphological, biochemical, and functional outcomes as compared to humans following SCI [17,90]. The moderately severe contusion injury (force setting 180 kdyn) results in partial deficits in hindlimb function in rats [91].

### 4.5. Ibrutinib Intraperitoneal (IP) Administration

Ibrutinib solutions were made by adding each solvent one by one in 5% DMSO, 40% PEG300, 5% Tween 80, and 50% saline, based on manufacturer recommendations for in vivo studies. Rats were randomly assigned to the following groups: (1) Sham operation without injury; (2) SCI; (3) SCI-injured rats received daily I.P. injections of 6 mg/kg/day of Ibrutinib for 1 week, beginning 3 h postinjury; (4) SCI-injured rats received daily I.P. injections of vehicle (5% DMSO, 40% PEG300, 5% Tween 80, and 50% saline) for 1 week, beginning 3 h postinjury; (5) SCI-injured rats received daily I.P. injections of 6 mg/kg/day of Ibrutinib for 2 weeks, beginning 3 h postinjury; (6) SCI-injured rats received daily I.P. injections of vehicle for 2 weeks, beginning 3 h postinjury. 

The Ibrutinib dosage is based on the dose used for chronic lymphocytic leukemia (therapeutic dose range 10–40 mg/kg/day for rats compared to 420 to 560 mg once daily for humans).

Lowering the Ibrutinib dose (6 mg/kg/day for rats) in this study was used to reduce the risk of side effects (bleeding). Lowering the dose of ibrutinib has clear potential to reduce the bleeding side effects of ibrutinib [92].

The starting time of intervention, beginning at 3 h post-injury, was chosen based on our recent study [17]. The therapeutic window will be further evaluated in future studies. The 1-week Ibrutinib treatment duration was chosen to target the acute microglia/macrophage activation after contusive SCI in rats [6], while the 2-week Ibrutinib treatment duration was designed to target the time course of the proliferation of astrocytes and B cells after contusive SCI in rats [17]. In mice, B cell proliferation peaks at 14 days and remains elevated at 28 days following contusive SCI [24].

### 4.6. Assessment of Locomotor Function

Open-field locomotor function was evaluated pre-injury, immediate, 3, and 7 days post-injury, and then weekly from until 4 or 11 weeks post-injury using the Basso, Beattie, and Bresnahan (BBB) rating scale [93] as in our previous studies [17,94]. Two evaluators, trained and certified by the Ohio State program, participated in the assessment in a blinded manner.

### 4.7. Monitoring Bladder Infection and Bleeding

Bladder infections were measured by examining urine color (green-yellow) and Transparency (cloudy). Rats were treated prophylactically with Cefazolin for the one week following injury to minimize the risk of bladder and other infections. Animals were checked twice a day for first 3 weeks, then twice a week, including body weight, bladder infection, bleeding, general activity, and breaths. Presence and extent of blood in urine were recorded. Bleeding was also evaluated by examining the hemorrhage at the lesion site, and by inspecting for bleeding/bruises in the eyes, forelimbs, and hindlimbs.

### 4.8. Spinal Cord Tissue Processing

For Western blot analysis, animals were euthanatized at 2, 7, or 14 days post-injury by Fatal Plus containing pentobarbital (100 mg/kg for rats, I.P. injection, *n* = 4 per group). A 5-mm spinal cord centered on the lesion site was removed and snap-frozen on dry ice, then stored at −80 °C. For histological staining analysis, at the conclusion of the locomotor assessment, animals were anesthetized and transcardially perfused with cold 0.1 M PBS, followed by 4% paraformaldehyde in phosphate-buffered saline (*n* = 5 per group). The spinal cords were removed and post-fixed with the same fixative overnight. Fixed spinal cord blocks (2 cm in length) centered at the lesion epicenter were immediately dissected, post-fixed in the same fixative solution for 4 h at 4 °C, cryoprotected in 30% sucrose in phosphate-buffered saline at 4 °C. Spinal cords were serially cryosectioned at a thickness of 20 μm. Every fifth section (interval between 100 μm) was mounted onto each Fisherbrand Superfrost Plus slide. The interval between two sections on each slide is 1 mm. Ten sets of slides were collected and stored at −20 °C.

### 4.9. Assessment of Lesion Volume, Total Tissue Sparing, White Matter Sparing, and Gray Matter Sparing

A modified histological eriochrome cyanine (EC) staining plus cresyl violet staining protocol for myelin that differentiates both white matter and cell bodies was performed to visualize spared spinal tissue in one set of slides, as described in our previous study [17]. Image analysis was performed on each EC-stained section and histological outcomes were evaluated by measuring total spinal section area, gray matter sparing, and lesion area on individual sections using Helo Axio Image System. Total tissue sparing, white matter sparing, gray matter sparing and lesion volume were analyzed from 11 evenly spaced sections as described in our recent study [17].

### 4.10. Western Blotting

Spinal cord protein samples were processed and analyzed using Western blotting as described in our previous study [17]. Briefly, the protein samples (60 μg of protein extract each sample) were loaded on SDS-PAGE gels and electrotransferred to nitrocellulose membranes. After transfer, membranes were incubated in blocking buffer (5% powdered milk in 1× TBS, 0.1% Tween 20) for 1 h at room temperature and incubated at 4 °C overnight with one of the primary antibodies. Blots were probed with a primary antibody against specific targets and reprobed with a secondary antibody against GADPH as a loading control. Blots were then incubated with IRDye anti-rabbit or anti-mouse secondary antibodies (1:5000). Blots were visualized and analyzed on the Li-Cor Odyssey infrared imaging system (Lincoln, NE, USA).

### 4.11. Double Immunofluorescence Confocal Imaging Analysis

Double immunofluorescence staining was performed as previously described [36]. Briefly, spinal cord cross-sections at the lesion site were incubated with an anti-mouse monoclonal antibody against Iba1 (GT10312, MA5-27726), 1:100; microglia/macrophage marker) or GFAP (GA5 mouse mAb #3670, CST) and a polyclonal antibody against phosphor-BTK (Tyr223, Tyr225) followed by incubation with Alexa Fluor 488 or 594-conjugated goat-anti rat secondary antibody. Using a laser scanning confocal microscopy system (Nikon C2+, Melville, NY, USA), the fluorescent Ibal/pBTK co-localization signals within the spinal cord section in the lesion site were captured (magnification 100×). 

### 4.12. Flow Cytometry Analysis

Sham-operated and injured rats with the treatment of Ibrutinib or vehicle were euthanized at 1-week post-injury or sham operation. Spleen samples were collected and processed for measuring the population of splenic CD45RA+ B cells using PE mouse anti-rat CD45RA antibody (Cat# 551402, BD Bioscience) and flow cytometry. Immediately after deep anesthesia, the spleen samples were collected. The spleen samples were removed and minced in a 3.5 cm-dish with Hank’s Balanced Salt Solution (HBSS, Invitrogen), then transferred to a 50 mL-tube with HBSS. The splenic cell samples were then passed through 40-μm nylon cell strainer to obtain a single-cell suspension as previously described [17]. Red blood cells (RBCs) in the resulting splenic cells were lysed using RBC lysis buffer (eBioscience). After washing, the splenic cell samples were resuspended in 5 mL of RPMI 1640 (Invitrogen). Flow cytometry system (Sony SY3200, Cell Sorter, Sony Biotechnology iCyT, San Jose, CA, USA, using the Core Facility) was used to measure the populations of CD45RA+ B cells in the splenic cell samples using antibodies against CD45RA (BD Bioscience) according to the manufacturer’s instructions and our recent study [17]. The population of CD45RA-positive B cells was automatically calculated as the percentage of specific CD45RA-positive cells. 

### 4.13. Statistical Analysis

BBB Scores, histological results (lesion volume and tissue sparing data), Western blot measures, and Flow cytometry data were statistically analyzed using StatView (SAS Institute, Cary, NC, USA). Data were presented as mean ± S.E.M. Group differences were evaluated by repeated-measures ANOVA followed by the Bonferroni post hoc test (*p* < 0.05 was considered significant). The *t*-test was used to analyze differences between the two groups.

## Figures and Tables

**Table 1:** Injury Parameters. Values are mean ± SEM. No significant differences in impact force, displacement, and velocity were found between the Ibrutinib treatment and vehicle groups (*n* = 10 per group).

Treatment Group	Actual Force (kdyn)	Displacement (μm)	Velocity (mm/s)
SCI + Ibrutinib	183.00 ± 0.95	1095.00 ± 54.6	123.10 ± 1.69
SCI + Vehicle	184.20 ± 0.96	1241.30 ± 88.19	121.60 ± 1.43

**Table 2:** Body Weight of Animals Per Week (G). Values are Mean ± SEM. At 1, 2, 3, 4 and 11 weeks, there are no significant differences in the bodyweight of animals per week were found between the vehicle- and Ibrutinib-treated animals (*n* = 10 per group); At 5, 6, and 7 weeks Ibrutinib treatment increased body weight of animals per week compared to vehicle-treated group. * Ibr: SCI + Ibrutinib treatment; Veh: SCI + Vehicle treatment.

Groups	1 W	2 W	3 W	4 W	5 W	6 W	7 W	8 W	11 W
Ibr *	266 ± 2.9	266 ± 2.8	272.3 ± 2.5	278 ± 2.3	290 ± 1.8 *	301 ± 3.1 *	312 ± 4.8 *	321 ± 6.1	340 ± 9.9
Veh	259 ± 5.3	261 ± 6.9	263.3 ± 5.6	268 ± 5.2	276 ± 4.8	286 ± 5.5	294 ± 6.6	305 ± 6.9	317 ± 9.8

## Data Availability

All data are published in this journal.
